# Bilateral Basal Ganglia Calcifications Manifesting as Psychosis With Manic Features: A Case Report on Fahr’s Syndrome

**DOI:** 10.7759/cureus.34547

**Published:** 2023-02-02

**Authors:** Aa'Mani C Dennis, Christian Nwabueze, Fahima Banu, Carolina D Nisenoff, Tolu Olupona

**Affiliations:** 1 Psychiatry, Interfaith Medical Center, Brooklyn, USA

**Keywords:** basal ganglia calcifications, psychosis, fahr’s syndrome, fahr’s disease, fahr’s disease or fahr’s syndrome, first episode psychosis, psychosis in elderly, bilateral ganglia calcifications, secondary psychosis

## Abstract

Fahr’s syndrome is a rare neurodegenerative disorder characterized by symmetric bilateral calcifications in the basal ganglia. While this is largely a hereditary disease with autosomal dominant inheritance, a small percentage is sporadic in nature with no metabolic or other underlying causes identified. Fahr’s syndrome has both neurological and psychiatric manifestations that include movement abnormalities, seizures, psychosis, and depression. Approximately 40% of patients with basal ganglia calcification present with psychiatric symptoms including mania, apathy, or psychosis.

We present a case of a 50-year-old woman with no previous medical or psychiatric history who presented with an altered mental status that progressed to psychosis over three years. On one admission, the patient was found to have elevated liver enzymes and a positive antinuclear antibody panel but was without electrolyte abnormalities or movement disturbances. The patient was subsequently diagnosed with unspecified psychosis in the emergency department, which was later revised to Fahr’s syndrome confirmed by neuroimaging. This report discusses her presentation, clinical symptoms, and management of Fahr’s syndrome. Above all, it underscores the importance of complete workup and adequate follow-up of middle-aged and elderly patients with cognitive and behavioral disturbances, as Fahr's syndrome can be elusive in the early stages.

## Introduction

Fahr’s syndrome is a rare, progressive, neurological condition that affects individuals in the fifth or sixth decade of life [1,2]. It is characterized by calcifications in the basal ganglia and is accompanied by neuropsychiatric symptoms. The pathogenesis is hypothesized to be due to a defective blood-brain barrier [2,3]. This abnormality allows excessive amounts of calcium and phosphorus to precipitate in the brain resulting in calcifications. In most cases of Fahr’s syndrome, the deposits are found in the basal ganglia [1-3]. However, they may also appear in the thalamus, cerebral cortex, and cerebellar subcortical matter [2]. In other cases, asymptomatic bilateral intracerebral calcifications are often discovered incidentally on computed tomography (CT) scans in approximately 15-20% of patients and are thought to be a result of normal aging [3]. Therefore, bilateral basal ganglia calcifications still require the presence of neuropsychiatric dysfunction to make a diagnosis of Fahr’s syndrome.

The etiology of Fahr’s disease could be primary or secondary. In the primary disease, known as Fahr’s disease, the etiology is unknown and the brain calcification is familial (Fahr’s disease). This type has an autosomal dominant inheritance and is associated with four different mutations (SLC20A2, PDGFRB, PDGFB, and XPR1) [2,3]. Secondary forms, denoted by the term “syndrome,” may be associated with systemic lupus erythematosus (SLE), metabolic causes such as hypoparathyroidism, brain infections, and exposure to toxic substances, or may be idiopathic.

This syndrome can present differently in each patient and there is no definitive correlation between the areas of the brain involved and the clinical presentation [2]. Generally, the presentation consists of neurologic signs such as seizures, gait disturbances, cognitive decline, bradykinesia, and resting tremors. Approximately 40% of patients may have psychiatric manifestations such as psychosis, anxiety, apathy, or mania [4,5].

Fahr’s syndrome is typically suspected after obtaining a brain CT scan indicating basal ganglia calcifications, after which routine labs are conducted. Laboratory values are typically within normal limits in patients with Fahr’s disease (primary familial brain calcification). Any abnormalities in routine laboratory tests should raise concerns for secondary causes and should be followed up with additional testing. Additional investigations include complete blood count (CBC) with differential, complete metabolic panel (CMP), serum calcium, phosphorus, magnesium, alkaline phosphatase (ALP), calcitonin, vitamin D, thyroid-stimulating hormone (TSH), parathyroid hormone (PTH), and cerebrospinal fluid (CSF) analysis if indicated [2].

## Case presentation

In 2019, a 50-year-old female with no past medical or psychiatric history presented to the emergency department with disorganized behavior, for which emergency medical services (EMS) were activated by her colleagues. She was uncooperative with staff in the emergency room, but not aggressive. Standard evaluation for potential causes of psychosis was initiated, including recommended laboratory tests, chest X-ray, CT imaging of the head, and electrocardiogram (EKG). Laboratory analysis consisted of CBC, CMP, calcium, vitamin B12 levels, urinalysis (UA), urine drug screen, blood alcohol level (BAL), and TSH. Laboratory testing (Table 1) showed mild elevation of aspartate aminotransferase (AST) and alanine aminotransferase (ALT). UA was positive for a few white blood cells (WBCs), red blood cells (RBCs), leukocyte esterase, ketones, and proteins, indicating a mild urinary tract infection. CBC was suggestive of iron deficiency anemia. The urine drug screen was negative for alcohol, amphetamines, barbiturates, benzodiazepines, cannabinoids, cocaine, methadone, and opiates. Vitamin B12 levels, calcium, blood urea nitrogen (BUN), and ammonia were all within normal range. Chest X-ray and EKG were both normal. CT imaging of the head was unremarkable and showed no acute changes, hemorrhages, ventriculomegaly, or calcifications. She was managed as a case of delirium of unknown etiology and discharged home after treatment with midazolam 5 milligrams (mg) via intramuscular injection, psychiatric review, and more than 24 hours of observation.

**Table 1:** Results of laboratory tests during three episodes of hospitalization over a period of three years

	Initial presentation	Two years after the initial presentation	Three years after the initial presentation	Normal range
White blood cell (WBC)	10.1	7.98	6.3	4.5-11.0
Hemoglobin (HGB)	11.7	12.7	10.8	12.0-16.0
Sodium	140	137	136	136-145
Potassium	4.8	4.6	4.2	3.5-5.0
Calcium	9.8	9.4	9.2	8.5-10.2
Chloride	102	98	105	96-106
Bicarbonate	22	22	23	20-28
Blood urea nitrogen (BUN)	18.0	17	16.6	6.0-23.0
Creatinine	0.83	0.92	0.82	0.50-1.20
Alanine aminotransferase (ALT)	41	21	17	0-33
Aspartate aminotransferase (AST)	91	16	14	5-32
Alkaline phosphatase (ALP)	83	131	72.9	35-104
Thyroid-stimulating hormone (TSH)	2.14	Not checked	3.36	0.27-4.20

**Figure 1:**
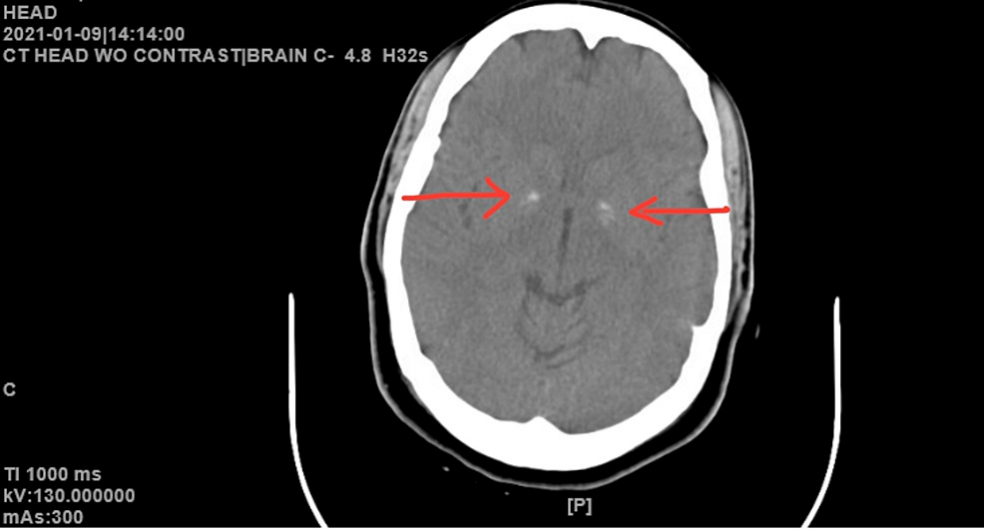
Axial CT without contrast scan of the brain taken in 2021. Red arrows indicate bilateral basal ganglia calcifications that are new in comparison to the 2019 imaging

During another emergency room evaluation in 2021, the patient presented to a different hospital after being picked up for wandering naked on the street. She had an acute change in mental status and portrayed psychotic features, including delusional ideations, visual hallucinations, and ideas of reference. The patient reported that she saw her pastor show up on her phone as a “ghost” and “things were falling down and disappearing all over the place.” Labs were unremarkable. CT scan, at this time, revealed new bilateral calcifications in the basal ganglia (Figure 1). At the time of this presentation, the bilateral basal ganglia calcifications were assumed to be noncontributory and considered the result of the normal aging process. She was diagnosed with unspecified psychosis based on the brevity of the symptoms. She was treated with intramuscular haloperidol 10 mg to manage symptoms of psychosis and was instructed to follow up with the partial hospitalization program (PHP) as an outpatient. Following discharge, she was prescribed haloperidol 10 mg by mouth and she continued with PHP via telebehavioral health. She was inconsistent in her attendance to the program and non-compliant with medications for several months. During several PHP sessions, the patient complained of severe anxiety and new-onset stuttering, poor appetite, difficulty sleeping, and subjective difficulty ambulating. Her inconsistent adherence to treatment continued into the next year.

In 2022, the patient presented with aggressive behavior and poor sleep for about two to three hours per night for one week. She appeared paranoid, suspicious of medications, and grandiose, stating “I am a famous American athlete. I worked at JP Morgan.” She was admitted for the management of unspecified psychosis. Standard evaluation for potential causes of psychosis was initiated, including testing for HIV, ammonia, copper, and ceruloplasmin. These tests were conducted to exclude organic causes of psychosis such as infection, electrolyte derangement, vitamin deficiency, substance use, endocrine dysfunction, or mineral imbalance. Additionally, an antinuclear antibody (ANA) comprehensive panel and rapid plasma reagin (RPR) were done to rule out psychosis due to SLE and syphilis. Labs were significant for elevated rheumatological markers for anti-ribonucleoprotein (RNP) antibodies, antiscleroderma-70 antibodies, and anti-centromere B antibodies (Table 2). The urine drug screen was negative. All other tests, including serum calcium, chest X-ray, and EKG, were unremarkable. Unfortunately, the patient refused head CT imaging on this visit.

**Table 2:** Rheumatological panel indicating positive markers for ribonucleoprotein, anti-scleroderma-70, and anti-centromere antibodies Anti-dsDNA antibodies = anti-double-stranded deoxyribonucleic acid antibodies; Anti-RNP = anti-ribonucleoprotein antibodies; Anti-Sm = anti-Smith antibodies; Anti-Scl70 antibodies = antiscleroderma-70 antibodies; Anti-SS-A antibodies= anti-Sjogren's syndrome-A antibodies; anti-SS-B antibodies = anti-Sjogren's syndrome-B antibodies; Anti-JO-1 antibodies = anti-histidyl tRNA synthetase antibodies.

Erythrocyte sedimentation rate (ESR)			
	Collection time: 07/30/22, 6:40 AM		
Result		Value	Ref. range
	Sedimentation rate	10	0-22 mm/hr
C-reactive protein (CRP)			
	Collection time: 07/30/22, 6:40 AM		
Result		Value	Ref. range
	CRP	<0.50 (L)	0.50-1.00 mg/dL
Antinuclear antibody (ANA) comprehensive panel			
	Collection time: 07/30/22, 6:40 AM		
Result		Value	Ref. range
	Anti-dsDNA antibodies	<1	0-9 IU/mL
	Anti-RNP antibodies	7.9 (H)	0.0-0.9 AI
	Anti-Sm antibodies	<0.2	0.0-0.9 AI
	Anti-Scl70 antibodies	1.1 (H)	0.0-0.9 AI
	Anti-SS-A antibodies	0.3	0.0-0.9 AI
	Anti-SS-B antibodies	<0.2	0.0-0.9 AI
	Antichromatin antibodies	0.2	0.0-0.9 AI
	Anti-JO-1 antibodies	<0.2	0.0-0.9 AI
	Anti-centromere B antibodies	>8.0 (H)	0.0-0.9 AI

Obtaining and reviewing previous information, including the CT scan, was integral in modifying the patient’s diagnosis to psychotic disorder due to Fahr’s syndrome (another medical condition) based on the bilateral basal ganglion calcifications on head CT, her history, and physical examinations that were inconsistent with any recognized mental health disorder, and the absence of any history or laboratory evidence of substance use. The patient was admitted for further psychiatric evaluation and management, where she was given risperidone 1 mg by mouth twice daily (BID) for psychosis. On the third day of admission, lithium 300 mg by mouth three times daily (TID) was added for mood stabilization and the antipsychotic was changed to oral fluphenazine 2.5 mg BID due to refractory psychotic symptoms. The patient became non-compliant with all psychiatric medications but opted to benefit from the milieu, group, and supportive therapy provided on the unit. She continued to show clinical improvement from admission until discharge. She became progressively calm and cooperative and participated in several group activity sessions. After 11 days, the patient reported her mood as "good” and denied suicidal and homicidal ideation, intent, or plan. Her psychotic and mood symptoms had resolved within a few days without medications, and she no longer had auditory and visual hallucinations. The patient expressed motivation to continue with mental health care as an outpatient. Her discharge medications included fluphenazine and lithium.

## Discussion

This patient’s presentation was characterized by psychotic and manic features that present similarly to that of schizophrenia and schizoaffective disorder and may be difficult to differentiate [6]. This case is a classic example of the sporadic type of Fahr’s syndrome in which the patient exhibited recurrent episodes of psychosis and mania characterized by visual hallucinations, delusional ideations, distractibility, impulsivity, and anxiety. The positive psychotic symptoms are not necessarily due to the classical dopamine-mediated manifestations that are seen in schizophrenia, although both Fahr’s syndrome and schizophrenia show some evidence of disruption in the cortex, particularly in the limbic system [6]. It appears, however, that some dopamine blockage results in the resolution of symptoms [7]. In a case of a 57-year-old male with Fahr’s syndrome with acute psychosis and manic features, the patient returned to baseline cognition following three days of treatment with haloperidol [7]. Similarly, this patient’s mentation and mood stabilized with just a few days of typical antipsychotic treatment and remained stable for the duration of her hospitalization. From this, we can only conclude antipsychotics and mood stabilizers are effective in stabilizing patients with Fahr’s syndrome who present with psychotic and manic features. There may be an opportunity to explore treatment responses to typical antipsychotics, as opposed to atypical antipsychotics in patients with Fahr's syndrome. Based on the efficacy of current antipsychotic medications, it can be inferred that continued compliance results in fewer hospitalizations and improved patient outcomes.

There are rare cases of psychosis associated with SLE. In a study of 485 participants with SLE, 2.3% were found to have psychosis that was due to lupus and 90% of those patients had positive rheumatological markers [8]. These markers include anti-double-stranded deoxyribonucleic acid (dsDNA), ANA, and or anti-Smith (anti-Sm) antibodies [8,9]. The markers in the above case were only weakly positive and included RNP antibodies, antiscleroderma-70, and anti-centromere B antibodies, which are consistent with mixed connective tissue disease, scleroderma diffuse type, and scleroderma calcinosis, Raynaud's phenomenon, esophageal dysmotility, sclerodactyly, and telangiectasia (CREST) type. There is no current research that supports correlations between psychosis and any of these autoimmune disorders. Therefore, these findings are likely independent of the development of psychosis and mania due to Fahr’s syndrome, as seen in this patient. Additionally, Fahr’s syndrome or episodic psychosis has not been correlated with mildly elevated liver enzymes or hepatitis. Furthermore, the patient’s ammonia and creatinine were within normal limits, excluding hepatic and renal injuries as the likely source of altered mentation. Hence, the elevated liver enzymes in this patient are considered to be unrelated to the development of symptoms. Also, the psychiatric manifestation in this patient could not have been due to traumatic brain injury-induced secondary psychosis, which is rare, and this patient has no history of traumatic brain injury [10].

Obtaining the head CT imaging with the first occurrence of acute change in mentation and ruling out other organic causes with standard workup and ancillary tests is a strength. The advantage of early CT imaging allowed our team to diagnose Fahr’s syndrome with subsequent imaging, noting the new bilateral basal ganglia calcifications. In most cases, this discovery is incidental and noncontributory, but in this case, it is significant in the progression of the patient’s symptoms. Adequate testing aided in excluding hypoparathyroidism, metabolic derangements, SLE, and other biological sources for altered mental status. Factors that may hinder the patient from favorable outcomes include poor insight, poorly treated psychiatric symptoms, multiple hospitalizations, noncompliance with medications, and low motivation for treatment. These are areas of improvement that may be addressed in outpatient treatment for adequate results.

## Conclusions

In summary, clinicians must consider both psychiatric and medical causes of acute mental status change in middle-aged patients. If the patient presents with multiple episodes and worsening symptoms, each episode of acute change should warrant a comprehensive laboratory workup and neuroimaging to rule out possible etiologies. Most non-organic psychiatric disorders are diagnosed in adolescence or young adulthood and are rare later in life. Oftentimes, initial episodes of psychosis presenting later in life have an organic cause. Therefore, initial presentations in middle-aged and elderly should be followed closely to determine the exact etiology of psychiatric symptoms. In this case, the patient presented with mild behavioral symptoms prior to developing basal ganglia calcifications. This suggests that Fahr's syndrome can be unpredictable and mild symptoms may precede the CT findings. A method of close follow-up that may prove beneficial can include serial CT imaging of the head and continued psychiatric evaluations. While these calcifications are often an incidental finding, in cases of psychosis, mood disturbances, and movement disorders presenting with these CT findings, the diagnosis of Fahr's syndrome should certainly be made. Due to the unpredictability in the presentation of Fahr’s syndrome, it is impossible to anticipate whether the presence of brain calcifications will be significant and if so, the type of neuropsychiatric or movement disorders they may cause. Thus, an accurate diagnosis of Fahr’s syndrome early on can only improve the management of new or worsening symptoms, should they arise. Patients with Fahr’s syndrome should be closely followed up with outpatient services and receive consistent symptom-based treatment to reduce relapse and hospitalizations.
